# Supplementary figures and images for: Alternate conformational trajectories in ribosome translocation
Source: PLoS Comput Biol. 2024 Aug 14;20(8):e1012319. doi: 10.1371/journal.pcbi.1012319 (PMC11346969; doi:10.1371/journal.pcbi.1012319)

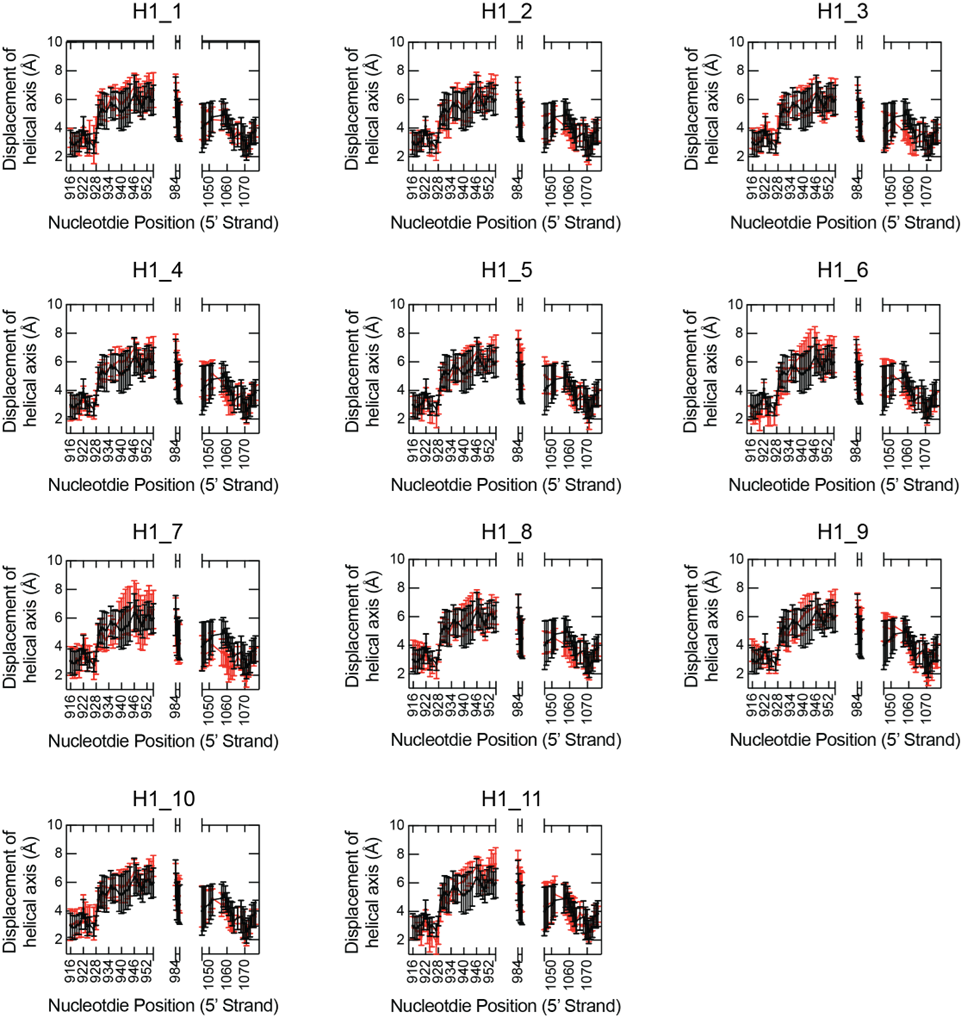

Supplement: S1 Fig — Displacement at each position along the calculated helical axis from the PRE state to the simulated conformation at 16° swivel for wild type (black) and top selected (red) hinge 1 variants. (TIF) [file pcbi.1012319.s001.tif]

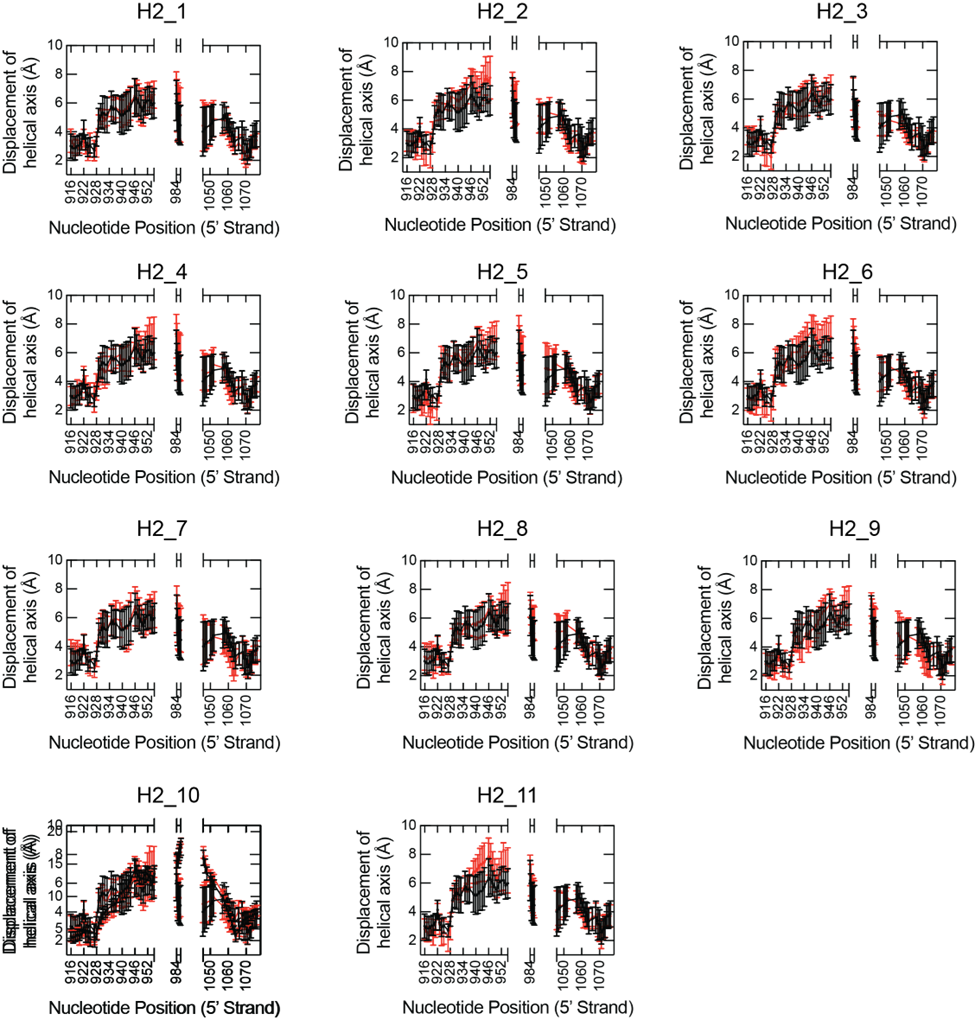

Supplement: S2 Fig — Displacement at each position along the calculated helical axis from the PRE state to the simulated conformation at 16° swivel for wild type (black) and top selected (red) hinge 2 variants. (TIF) [file pcbi.1012319.s002.tif]

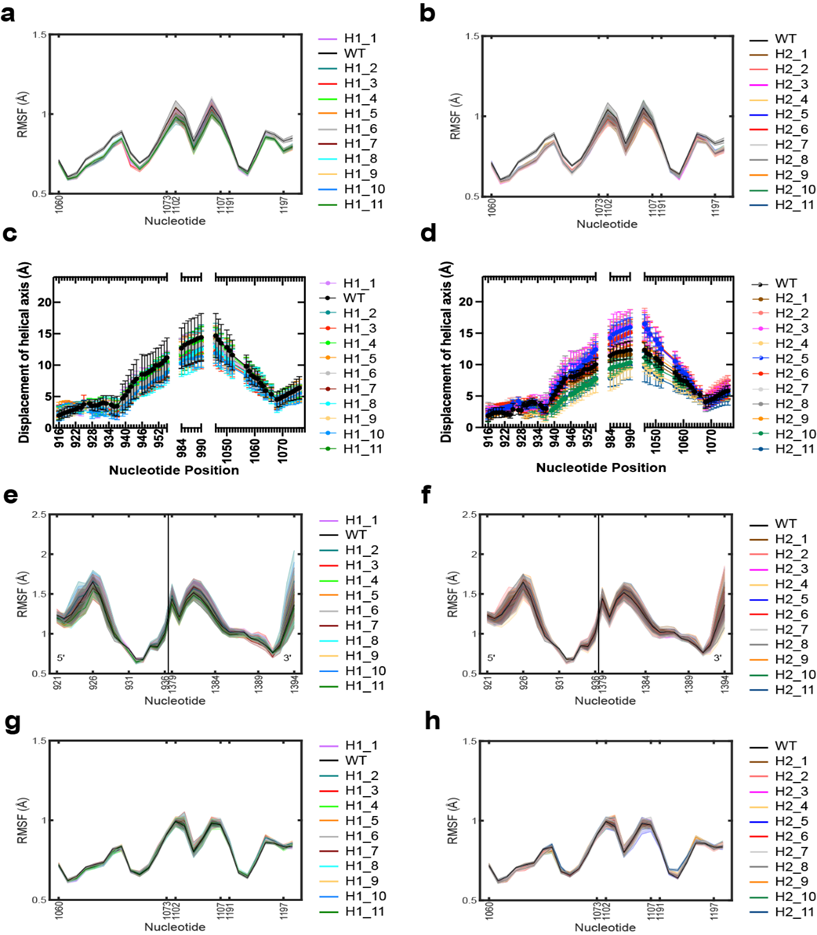

Supplement: S3 Fig — (a) Root-mean-square-fluctuations (RMSF) of hinge 2 during forward swivel for wild type and top selected hinge 1 variants. (b) Same as (a) for wild type and top selected hinge 2 variants. (c) Displacement at each position along the calculated helical axis from the maximum swivel (INT3) state to the intermediate (13° head swivel) conformation for wild type and top selected hinge 1 variants. (d) Same as (c) for wild type and top selected hinge 2 variants. (e) Root-mean-square-fluctuations (RMSF) of hinge 1 during reverse swivel for wild type and top selected hinge 1 variants. (f) Same as (e) for wild type and top selected hinge 2 variants. (g) Root-mean-square-fluctuations (RMSF) of hinge 2 during reverse swivel for wild type and top selected hinge 1 variants. (h) Same as (g) for wild type and top selected hinge 2 variants. (TIF) [file pcbi.1012319.s003.tif]

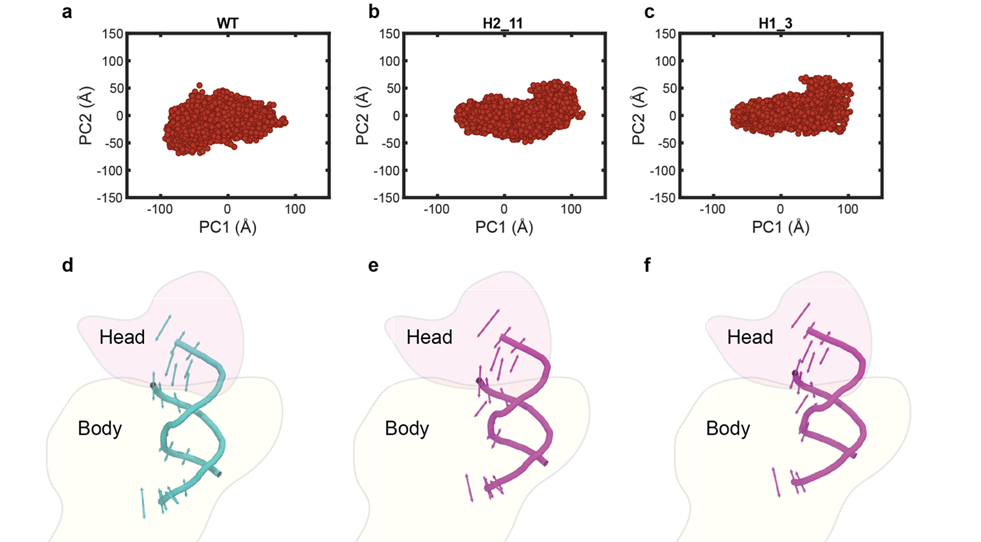

Supplement: S4 Fig — 2 dimensional projections of principal component 1 (PC1) and 2 (PC2) for (a) WT, and the (b) H2_11, (c) H1_3 hinge variants. Structural description of PC1 projected onto the structure of hinge 1 of the SSU for (d) WT, and the (e) H2_11, (f) H1_3 hinge variants. (TIF) [file pcbi.1012319.s004.tif]

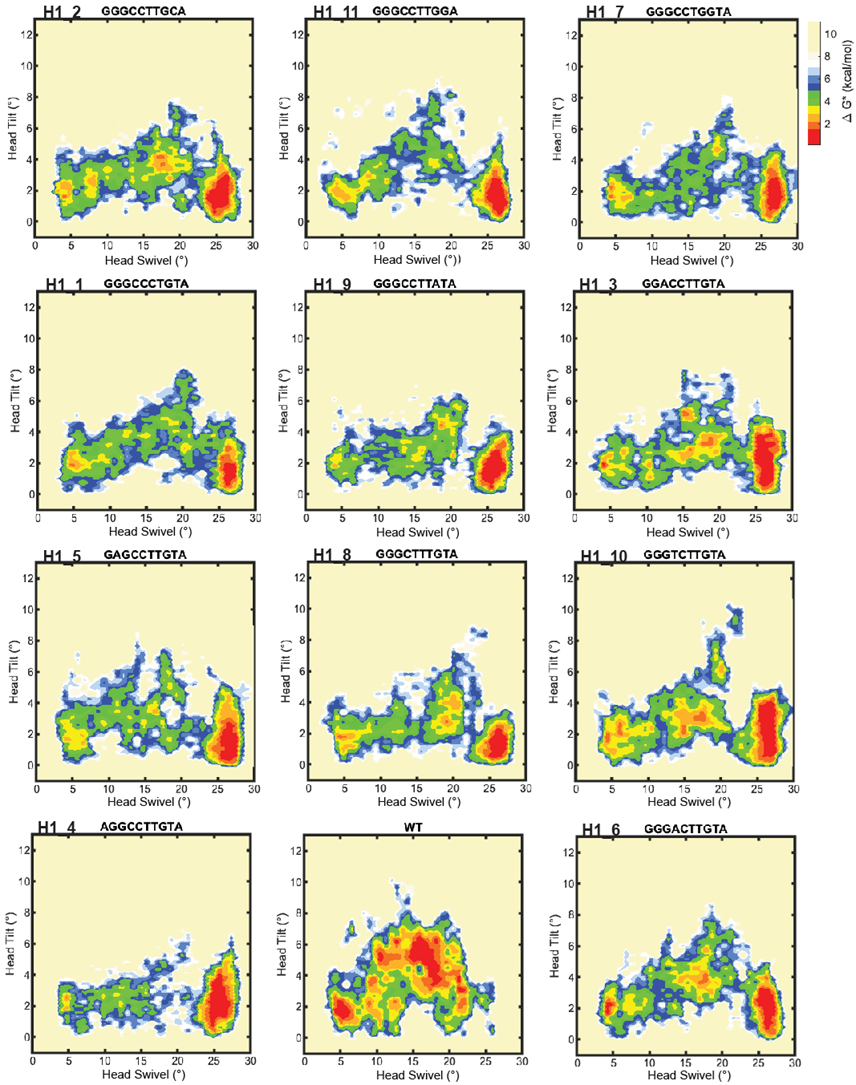

Supplement: S5 Fig — Relative free energy landscapes of the forward swivel (PRE to INT3) stage of translocation with head domain swivel and tilt angles as reaction coordinates. The heat maps are obtained by normalizing the number of frames in the targeted MD simulation, where the frame count is divided by the maximum frame count. Shown are the maps for the top 12 hinge 1 enriched genotypes. Maps correspond to ten trajectories per genotype. (TIF) [file pcbi.1012319.s005.tif]

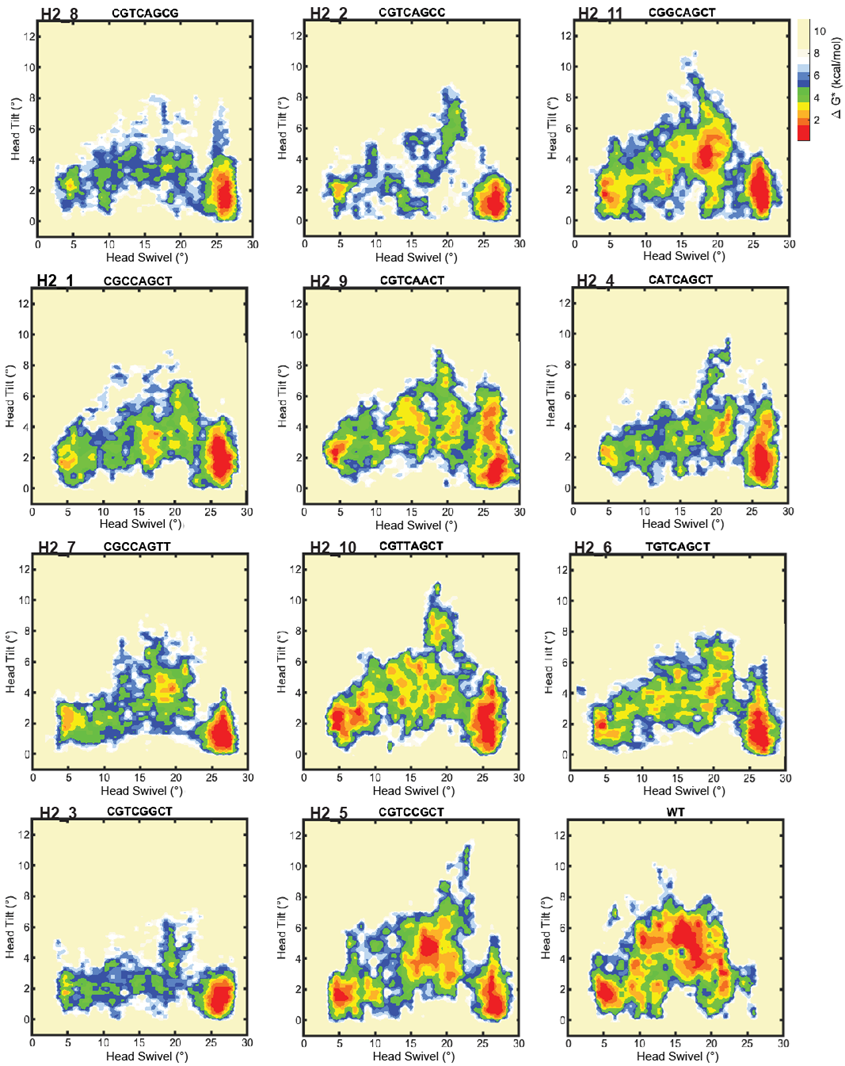

Supplement: S6 Fig — Relative free energy landscapes of the forward swivel (PRE to INT3) stage of translocation with head domain swivel and tilt angles as reaction coordinates. The heat maps are obtained by normalizing the number of frames in the targeted MD simulation, where the frame count is divided by the maximum frame count. Shown are the maps for the top 12 hinge 2 enriched genotypes. Maps correspond to ten trajectories per genotype. (TIF) [file pcbi.1012319.s006.tif]

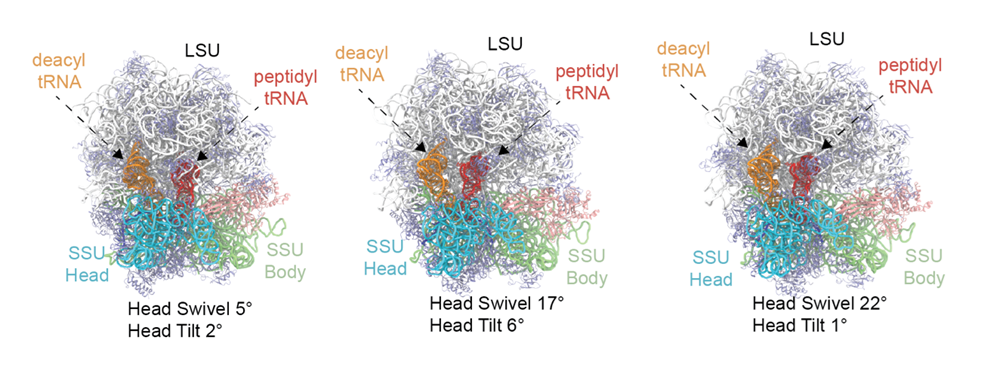

Supplement: S7 Fig — Simulated representative structures at different head-swivel and head-tilt angles during forward swivel motions of the ribosome. In the representation SSU head (cyan) undergoes swivel and tilt with respect to the SSU body (green). The positions of the peptidyl (red) and deacyl (orange) tRNA are also highlighted to demonstrate movement during forward head-swivel. The structures correspond to the PRE (left), INT2 (center) and INT3 (right) states. (TIF) [file pcbi.1012319.s007.tif]

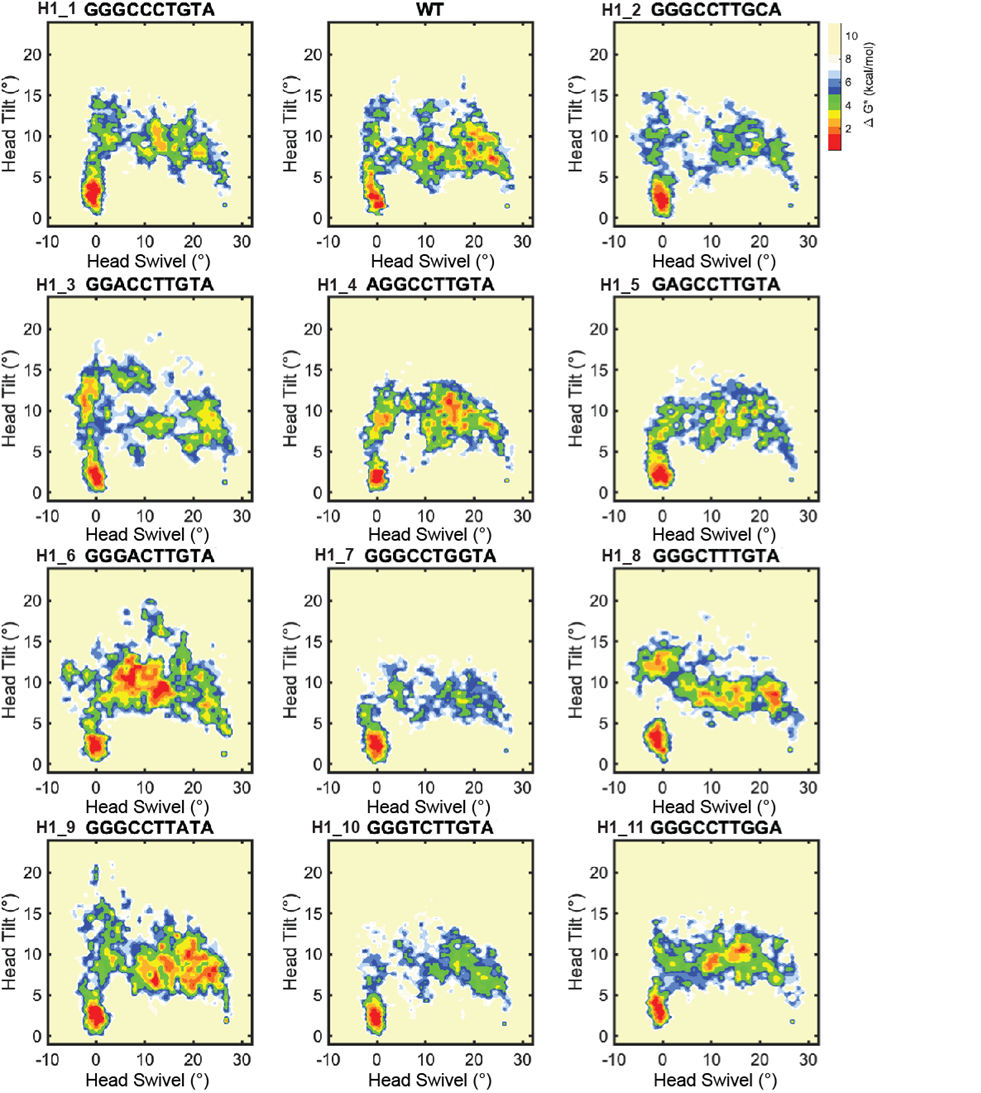

Supplement: S8 Fig — Relative free energy landscapes of the reverse swivel (INT3 to POST) stage of translocation with head domain swivel and tilt angles as reaction coordinates. The heat maps are obtained by normalizing the number of frames in the targeted MD simulation, where the frame count is divided by the maximum frame count. Shown are the maps for the top 12 hinge 1 enriched genotypes. Maps correspond to ten trajectories per genotype. (TIF) [file pcbi.1012319.s008.tif]

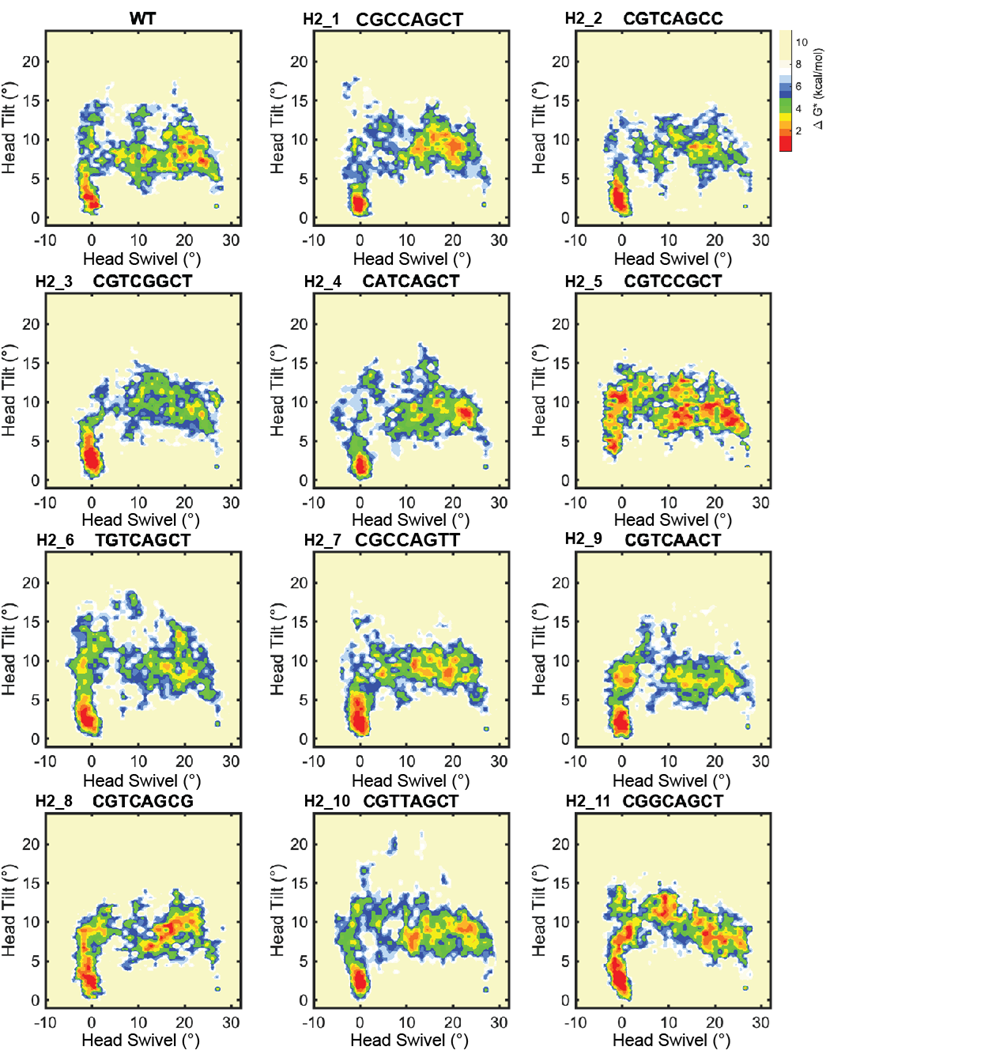

Supplement: S9 Fig — Relative free energy landscapes of the reverse swivel (INT3 to POST) stage of translocation with head domain swivel and tilt angles as reaction coordinates. The heat maps are obtained by normalizing the number of frames in the targeted MD simulation, where the frame count is divided by the maximum frame count. Shown are the maps for the top 12 hinge 2 enriched genotypes. Maps correspond to ten trajectories per genotype. (TIF) [file pcbi.1012319.s009.tif]

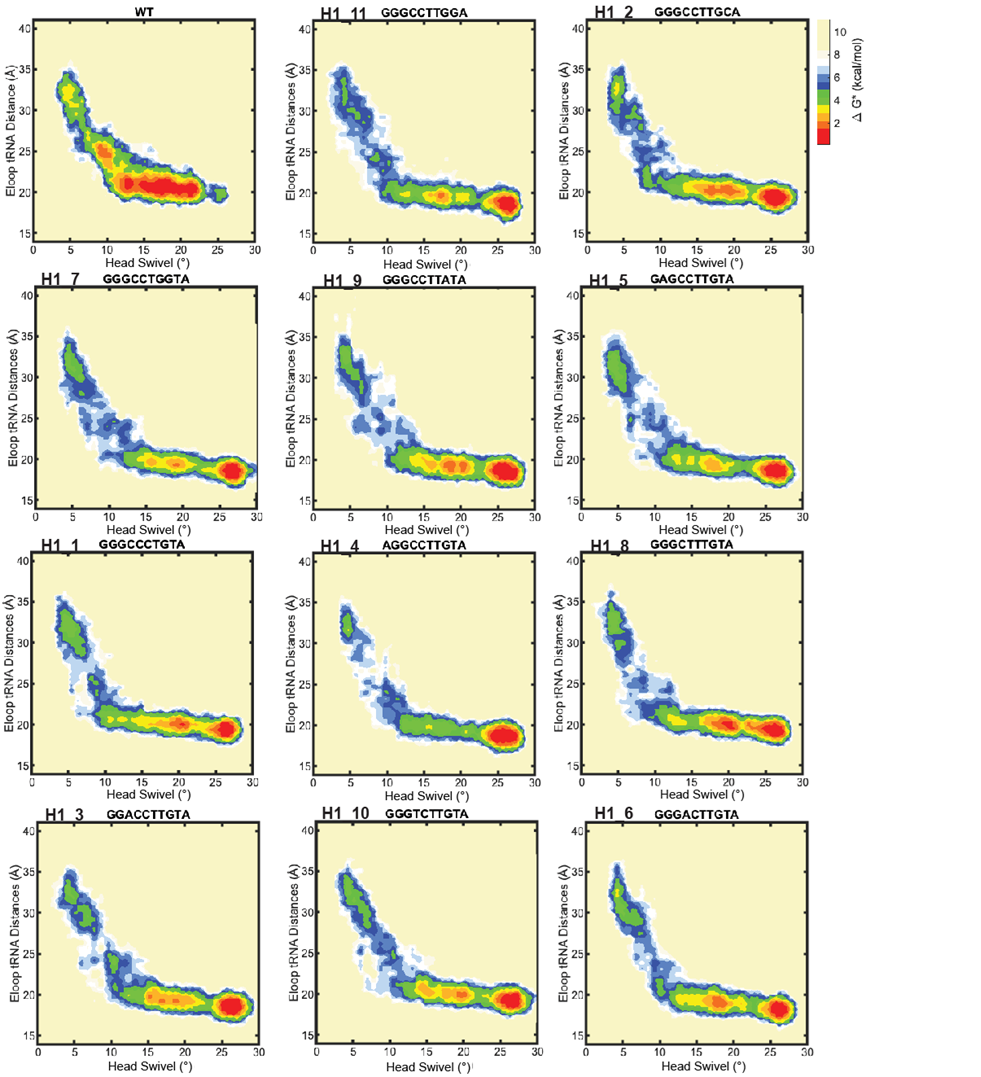

Supplement: S10 Fig — Relative free energy landscapes of the forward swivel (PRE to INT3) stage of translocation with head domain swivel angle and E loop-Deacyl tRNA distance as reaction coordinates. The heat maps are obtained by normalizing the number of frames in the targeted MD simulation, where the frame count is divided by the maximum frame count. Shown are the maps for the top 12 hinge 1 enriched genotypes. Maps correspond to ten trajectories per genotype. (TIF) [file pcbi.1012319.s010.tif]

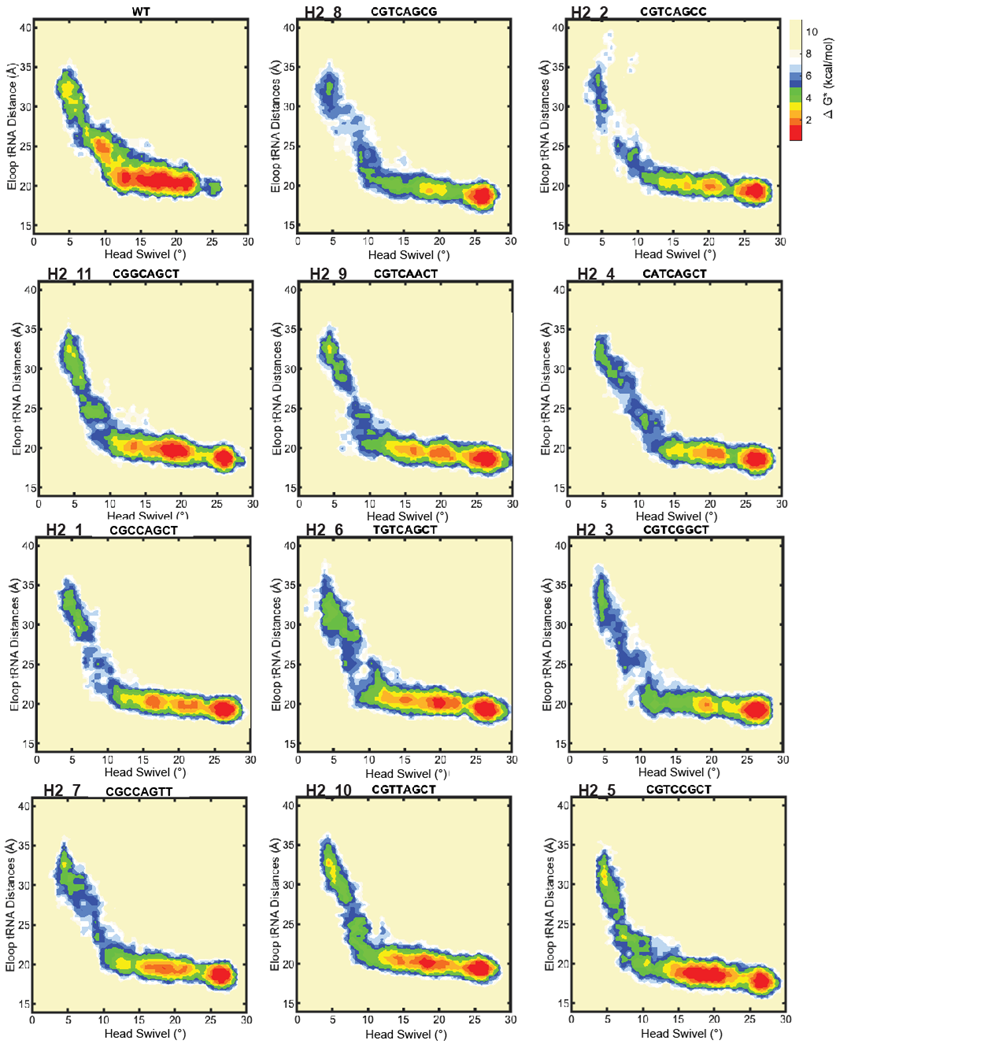

Supplement: S11 Fig — Relative free energy landscapes of the forward swivel (PRE to INT3) stage of translocation with head domain swivel angle and E loop-Deacyl tRNA distance as reaction coordinates. The heat maps are obtained by normalizing the number of frames in the targeted MD simulation, where the frame count is divided by the maximum frame count. Shown are the maps for the top 12 hinge 2 enriched genotypes. Maps correspond to ten trajectories per genotype. (TIF) [file pcbi.1012319.s011.tif]

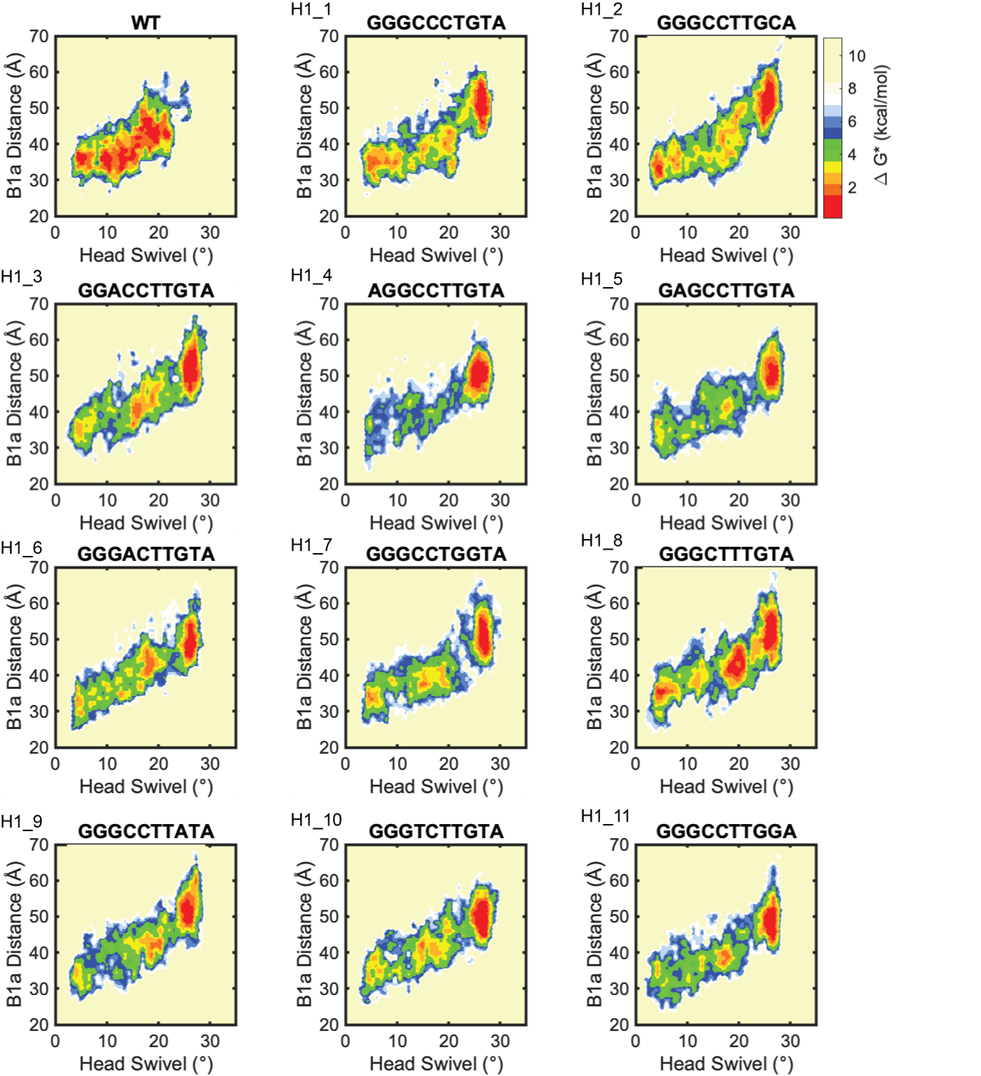

Supplement: S12 Fig — Relative free energy landscapes of the forward swivel (PRE to INT3) stage of translocation with head domain swivel angle and bridge B1a distance as reaction coordinates. The heat maps are obtained by normalizing the number of frames in the targeted MD simulation, where the frame count is divided by the maximum frame count. Shown are the maps for the top 12 hinge 1 enriched genotypes. Maps correspond to ten trajectories per genotype. (TIF) [file pcbi.1012319.s012.tif]

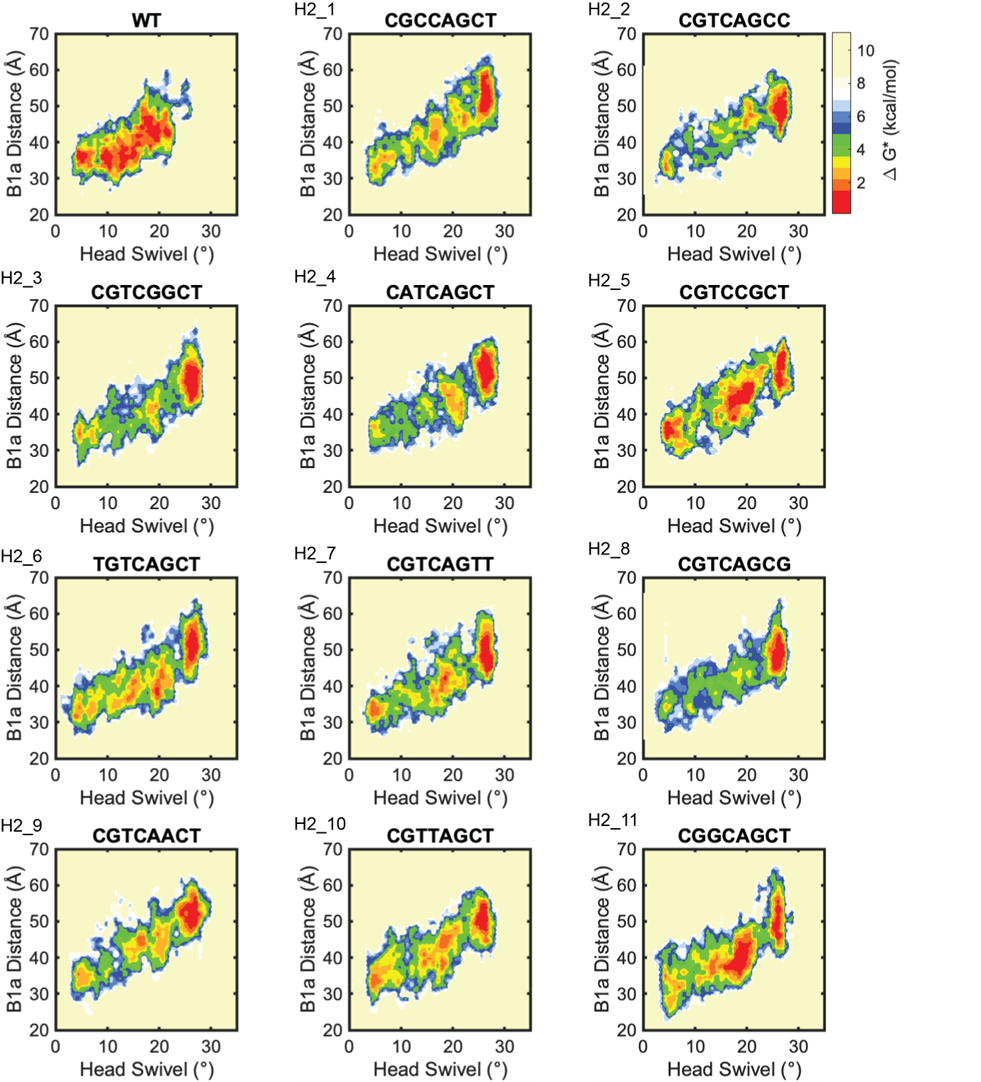

Supplement: S13 Fig — Relative free energy landscapes of the forward swivel (PRE to INT3) stage of translocation with head domain swivel angle and bridge B1a distance. The heat maps are obtained by normalizing the number of frames in the targeted MD simulation, where the frame count is divided by the maximum frame count. Shown are the maps for the top 12 hinge 2 enriched genotypes. Maps correspond to ten trajectories per genotype. (TIF) [file pcbi.1012319.s013.tif]

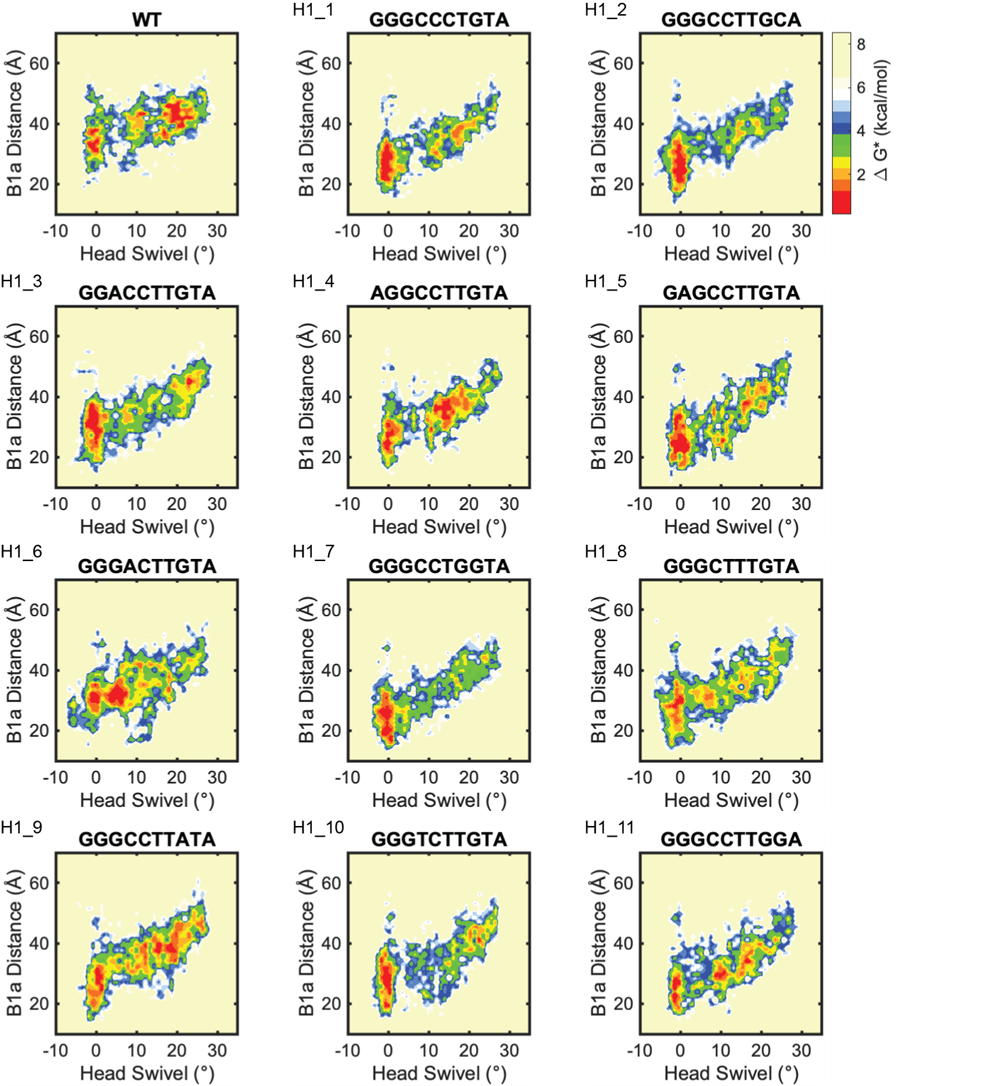

Supplement: S14 Fig — Relative free energy landscapes of the forward swivel (PRE to INT3) stage of translocation with head domain swivel angle and bridge B1a distance. The heat maps are obtained by normalizing the number of frames in the targeted MD simulation, where the frame count is divided by the maximum frame count. Shown are the maps for the top 12 hinge 1 enriched genotypes. Maps correspond to ten trajectories per genotype. (TIF) [file pcbi.1012319.s014.tif]

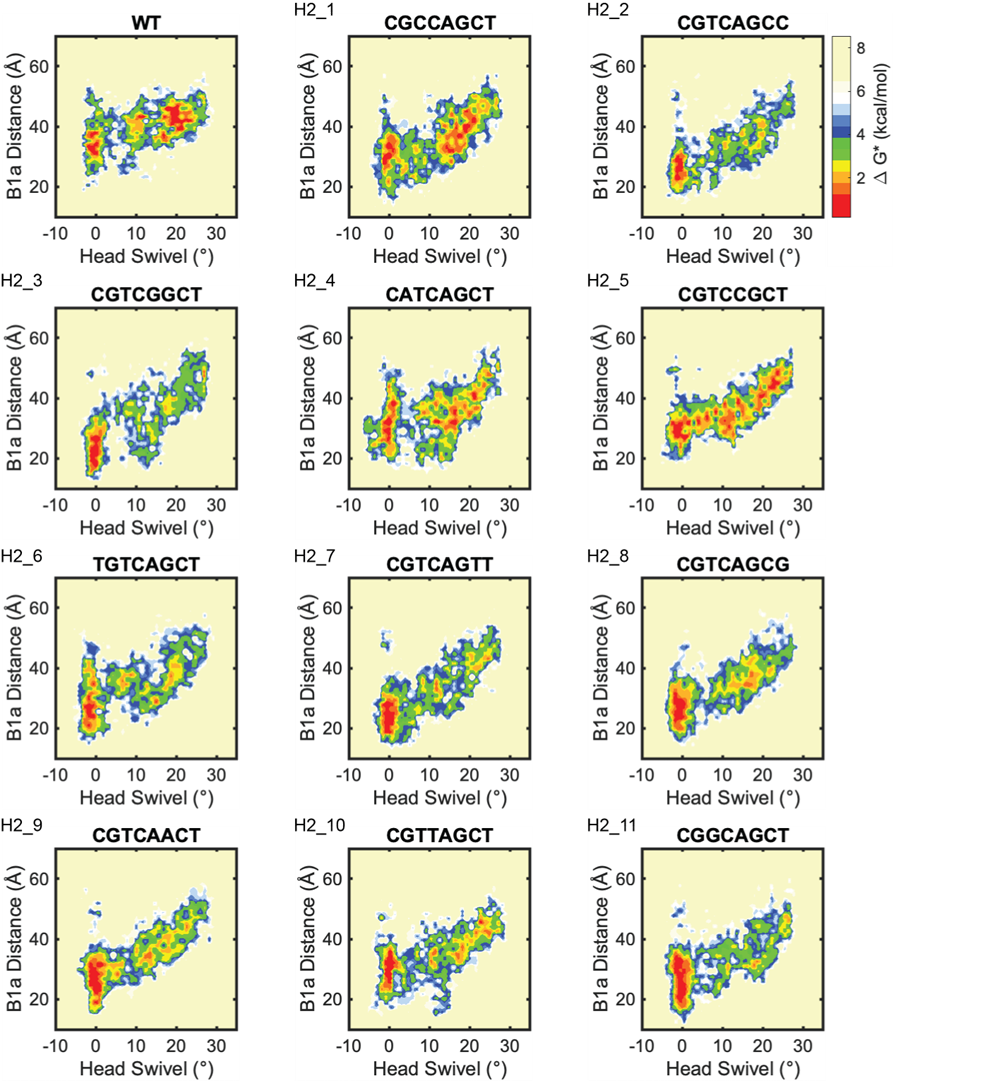

Supplement: S15 Fig — Relative free energy landscapes of the forward swivel (PRE to INT3) stage of translocation with head domain swivel angle and bridge B1a distance. The heat maps are obtained by normalizing the number of frames in the targeted MD simulation, where the frame count is divided by the maximum frame count. Shown are the maps for the top 12 hinge 2 enriched genotypes. Maps correspond to ten trajectories per genotype. (TIF) [file pcbi.1012319.s015.tif]

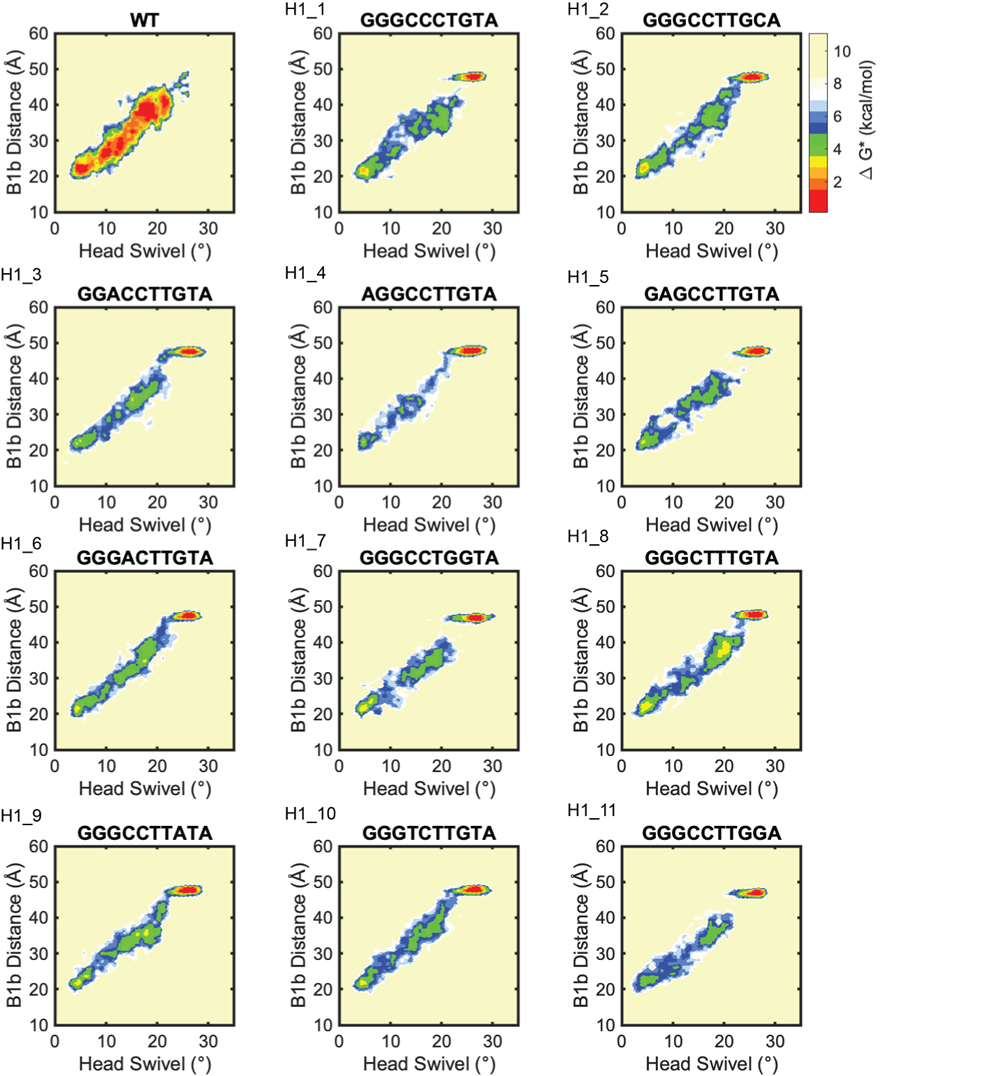

Supplement: S16 Fig — Relative free energy landscapes of the forward swivel (PRE to INT3) stage of translocation with head domain swivel angle and bridge B1b distance. The heat maps are obtained by normalizing the number of frames in the targeted MD simulation, where the frame count is divided by the maximum frame count. Shown are the maps for the top 12 hinge 1 enriched genotypes. Maps correspond to ten trajectories per genotype. (TIF) [file pcbi.1012319.s016.tif]

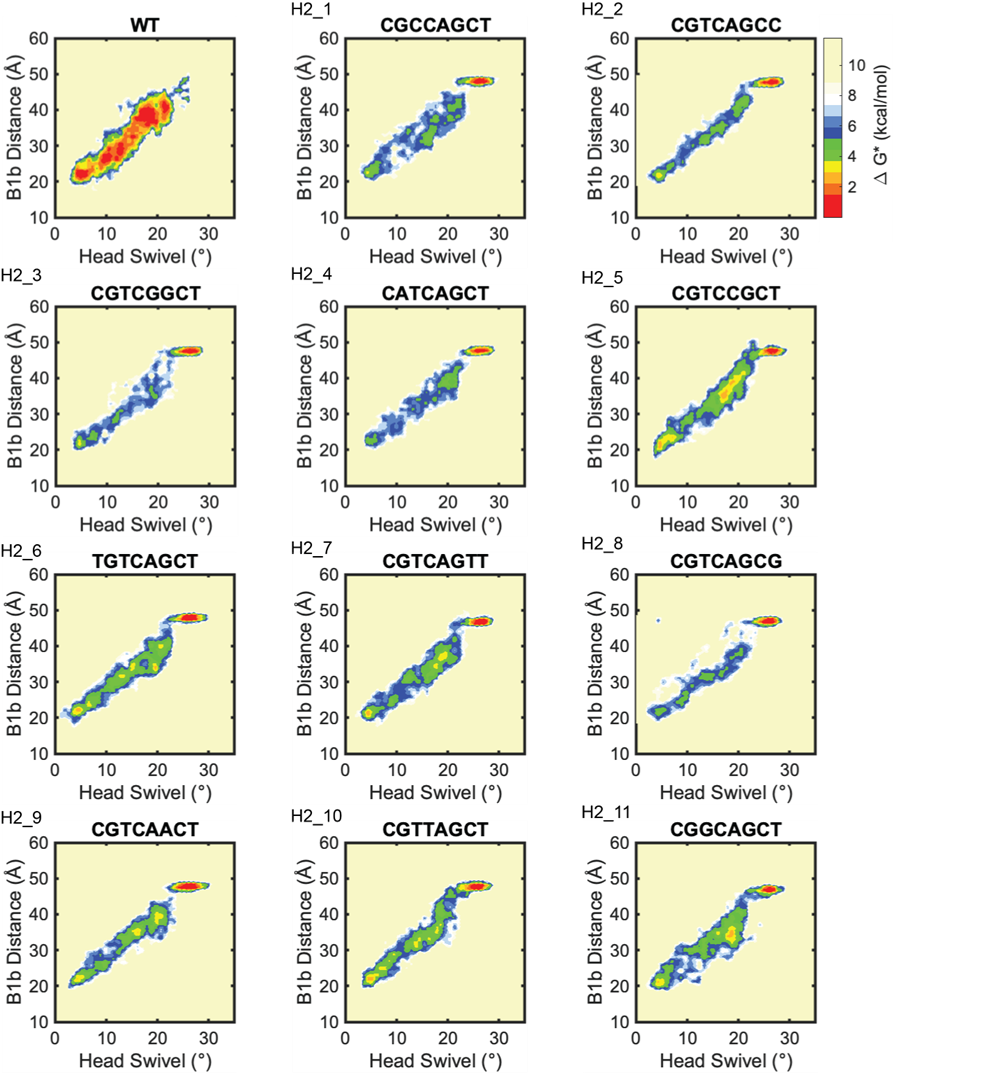

Supplement: S17 Fig — Relative free energy landscapes of the forward swivel (PRE to INT3) stage of translocation with head domain swivel angle and bridge B1b distance. The heat maps are obtained by normalizing the number of frames in the targeted MD simulation, where the frame count is divided by the maximum frame count. Shown are the maps for the top 12 hinge 2 enriched genotypes. Maps correspond to ten trajectories per genotype. (TIF) [file pcbi.1012319.s017.tif]

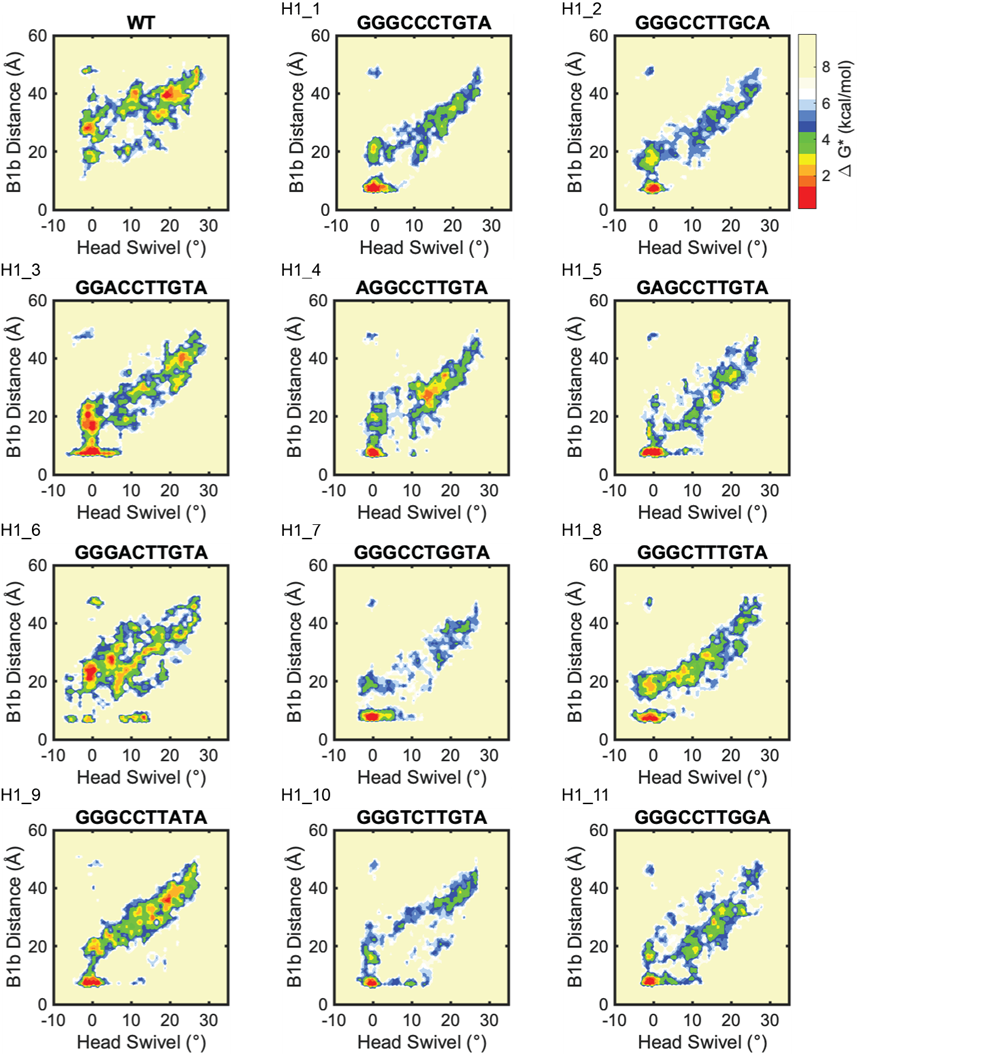

Supplement: S18 Fig — Relative free energy landscapes of the forward swivel (PRE to INT3) stage of translocation with head domain swivel angle and bridge B1b distance. The heat maps are obtained by normalizing the number of frames in the targeted MD simulation, where the frame count is divided by the maximum frame count. Shown are the maps for the top 12 hinge 1 enriched genotypes. Maps correspond to ten trajectories per genotype. (TIF) [file pcbi.1012319.s018.tif]

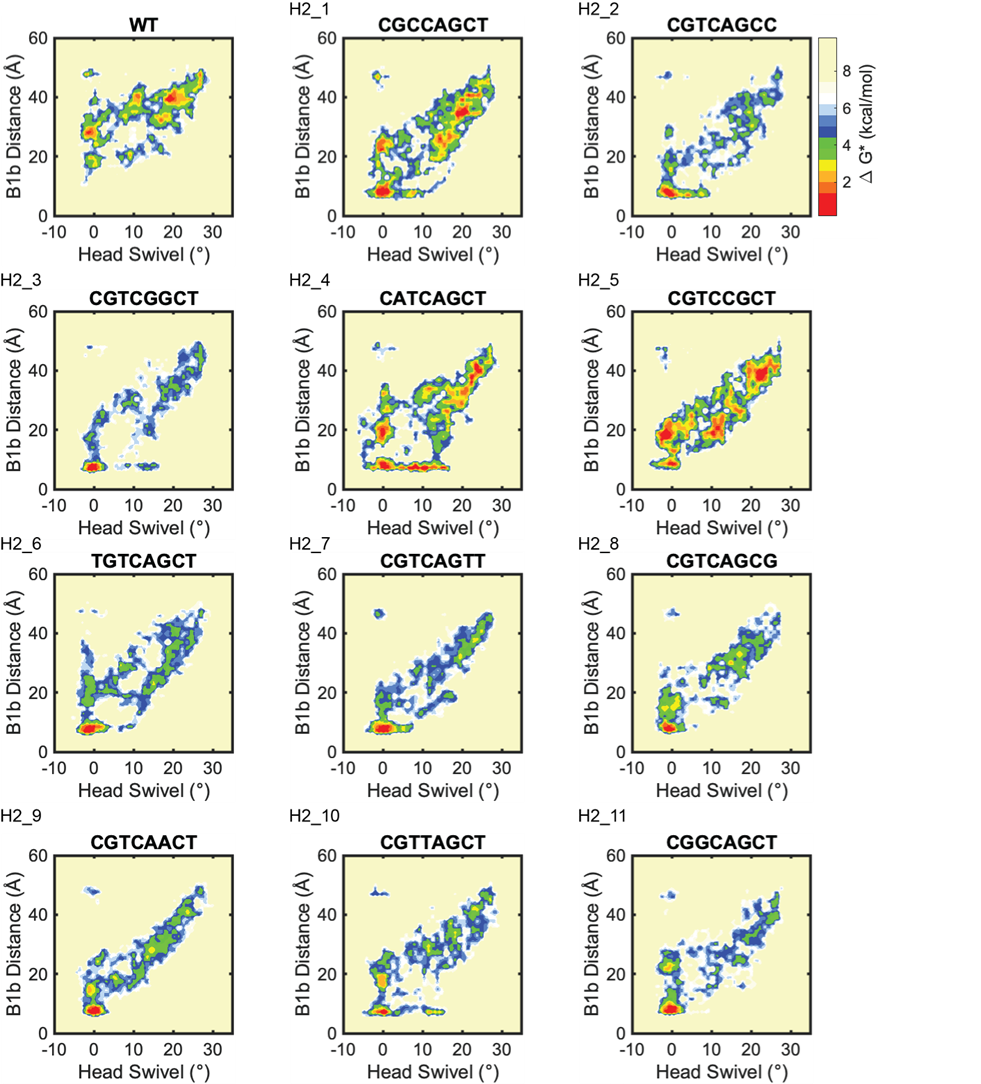

Supplement: S19 Fig — Relative free energy landscapes of the forward swivel (PRE to INT3) stage of translocation with head domain swivel angle and bridge B1b distance. The heat maps are obtained by normalizing the number of frames in the targeted MD simulation, where the frame count is divided by the maximum frame count. Shown are the maps for the top 12 hinge 2 enriched genotypes. Maps correspond to ten trajectories per genotype. (TIF) [file pcbi.1012319.s019.tif]

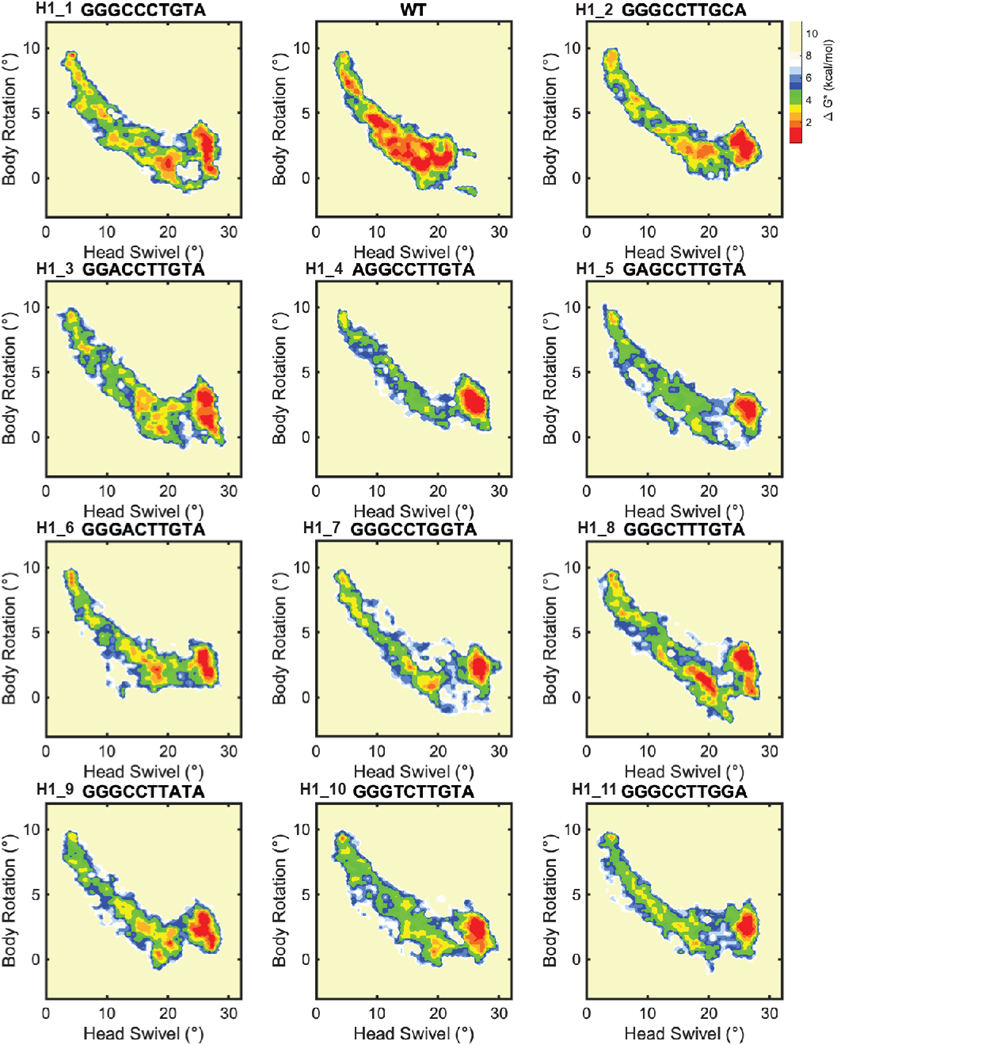

Supplement: S20 Fig — Relative free energy landscapes of the forward swivel (PRE to INT3) stage of translocation with head domain swivel angle and small subunit rotation as reaction coordinates. The heat maps are obtained by normalizing the number of frames in the targeted MD simulation, where the frame count is divided by the maximum frame count. Shown are the maps for the top 12 hinge 1 enriched genotypes. Maps correspond to ten trajectories per genotype. (TIF) [file pcbi.1012319.s020.tif]

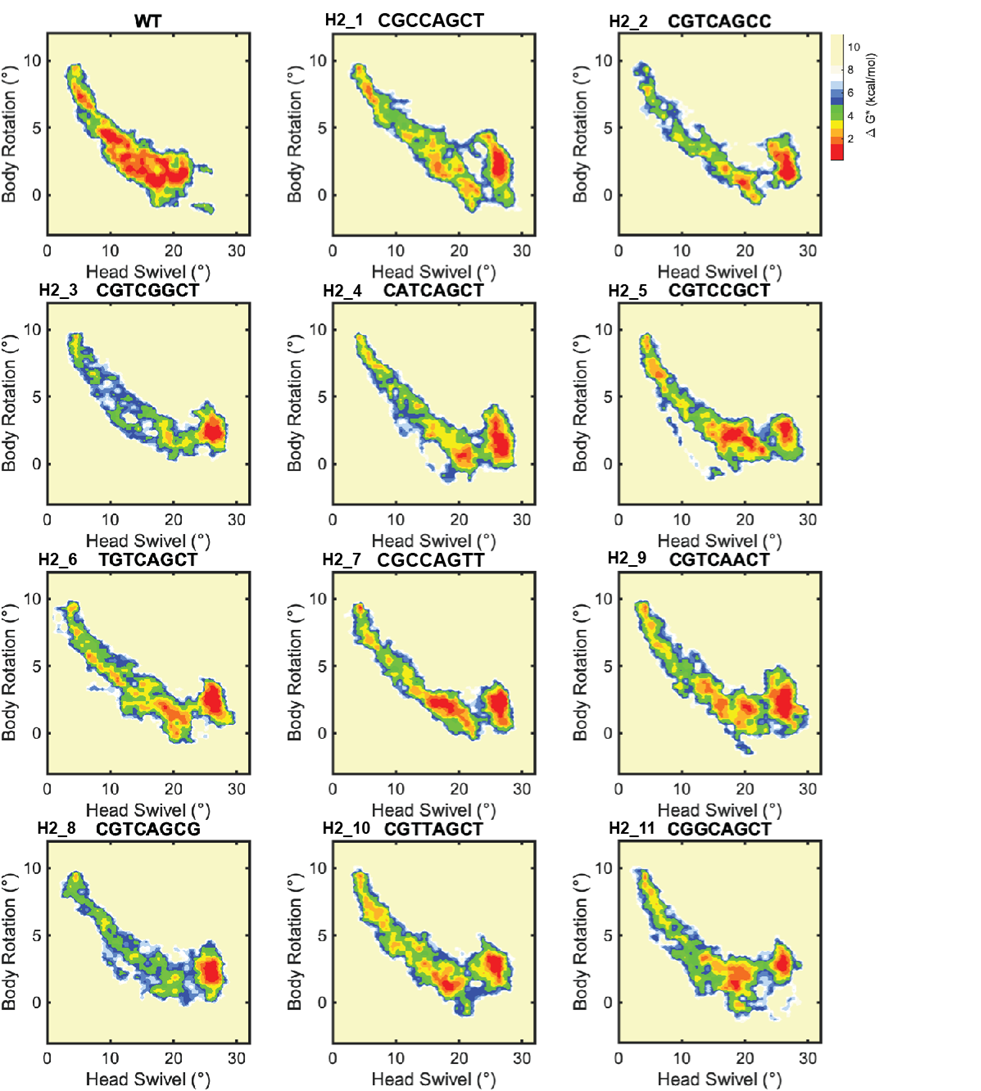

Supplement: S21 Fig — Relative free energy landscapes of the forward swivel (PRE to INT3) stage of translocation with head domain swivel angle and small subunit rotation as reaction coordinates. The heat maps are obtained by normalizing the number of frames in the targeted MD simulation, where the frame count is divided by the maximum frame count. Shown are the maps for the top 12 hinge 2 enriched genotypes. Maps correspond to ten trajectories per genotype. (TIF) [file pcbi.1012319.s021.tif]
